# *Trichomonas vaginalis* strain diversity among female sex workers in Ecuador using DNA sequence-based typing

**DOI:** 10.1186/s12879-025-12185-7

**Published:** 2025-12-04

**Authors:** Claire Elizabeth Broad, Luz Marina Llangari-Arizo, Kenneth Gordon Laing, Natalia Cristina Romero-Sandoval, Philip John Cooper, Syed Tariq Sadiq

**Affiliations:** 1 Institute for Infection and Immunity, St George’s School of Health and Medical Sciences, City St George’s, University of London, London, UK; 2 Escuela de Medicina, Universidad Internacional del Ecuador UIDE, Quito, Ecuador; 3 Unitat de Bioestadística Universitat Autónoma de Barcelona, Barcelona, Spain; 4 Red Internacional Grups de Recerca d’América i África Llatines-GRAAL, Barcelona, Spain; 5 Clinical Academic Group for Infection and Immunity, St George’s University Hospitals NHS Foundation Trust, London, UK

**Keywords:** Sexual networks, Sex workers, Trichomonas vaginalis, Sequence-typing, Ecuador

## Abstract

**Background:**

Molecular methods to track the spread of *Trichomonas vaginalis* (TV) infection, the most common curable non-viral sexually transmitted infection globally, associated with poor reproductive health outcomes and low socio-economic status are challenging, as ultra-long repetitive DNA sequences in TV make whole genome sequencing difficult. We undertook multilocus sequence typing (MLST) of TV using nested-PCR from clinical samples, to describe strain diversity among at-risk female sex-workers (FSWs) in Ecuador.

**Methods:**

Sociodemographic data and vulvo-vaginal swabs were collected from two groups of FSWs, street-based workers (SBWs) and brothel-based workers (BBWs). DNA extracts, positive for TV by real-time PCR, were amplified by two-step nested-PCR for seven TV genes and MLST-amplicon libraries sequenced using Illumina MiSeq. Sequence types (STs) were clustered into clonal complexes using *goeBURST* and population structure investigated using STRUCTURE.

**Results:**

Of 250 FSWs, 58 were positive for TV by real-time PCR. Subsets of TV positive vaginal DNA extracts were sequence-typed from 15 SBWs and 17 BBWs, alongside a non-sex worker sample collected from the same region, and a positive control. Compared with BBWs, SBWs were older (*p* < 0.001) and earnt less for sex work. TV-MLST revealed new STs and two major population subtypes. No associations were found between ST and behaviouralcharacteristics. *goeBURST* analysis of study samples identified four clonal complexes in which the largest complex comprised primarily of BBWs. When combined with a larger international dataset, *goeBURST* revealed 9 clonal complexes and 24 separate STs or nodes. FSWs with the same ancestral TV population structure were not displaced by the added STs.

**Conclusion:**

TV-MLST revealed high strain diversity among Ecuadorian FSWs and a two-type sub-population. The preservation of links between STs associated with some FSWs when adding a larger set of archived STs, suggests potential for use as an aid to TV associated sexual network identification.

**Supplementary Information:**

The online version contains supplementary material available at 10.1186/s12879-025-12185-7.

## Introduction

*Trichomonas vaginalis* (TV), the most common curable non-viral sexually transmitted infection (STI) globally, with the highest prevalence reported in lower- and middle-income countries [1, 2] is associated with poverty, sexual risk behaviours, older age, and altered vaginal microbiome [2, 3]. TV epidemiology has been linked to density of sexual networks in at-risk heterosexual populations such as sex workers [4, 5], where strains may be diverse or closely related in more closed networks between clients and sex workers, and perhaps where discrete sets of strains account for significant prevalence. Such closed networks lend themselves to interventions using genomic technologies targeting potential hotspots of transmission.

Conducting whole genome sequencing (WGS) analysis for TV, is challenging as it has a large genome size of ~ 160 Mb, approximately 38 Mb of which consists of long repetitive elements scattered throughout the genome [6], significant genomic variability, numerous gene families and limited reference genomes [7]. Consequently, genotyping methods such as multilocus sequence typing (MLST) and actin gene sequencing have been used [8, 9], often with in vitro culture to obtain pathogen DNA.

TV MLST schemes, including one that more recently utilised a modified nested-PCR approach directly from clinical samples, in order to bypass the need for culture [10], have described up to 47 diverse sequence types [11–13] and frequency differences of TV MLST loci have consistently identified two ancestral TV populations, termed Type 1 and Type 2. Type 1, has been described as the original ancestral clade, is associated with high parasitic load and found to be frequently infected with TV virus (TVV) [13], which possibly enhances pathogenicity and symptom severity [10]. Type 2 has been described as associated with treatment failure, with some data linking it to low parasitic load [11, 12].

We recently investigated STI prevalence among brothel and street based female sex workers (FSWs) in northwest Ecuador, identifying over 20% prevalence of TV from vulvo-vaginal swab samples (VVS), that was disproportionately associated with being a street-based worker (SBW) [14]. In this analysis, we hypothesized that TV MLST may have potential to identify any links between TV strains among FSWs.

## Methods

Full details regarding methodologies and results in relation to other STIs have been described previously [14]. Briefly, 205 brothel workers (BBWs), 45 SBWs and 250 non-sex workers (NSWs) were recruited from a village, town and city (A, B and C respectively) in north-west Ecuador. DNA was extracted from vulvovaginal swabs and transferred to the UK for STI testing by PCR and TV MLST analysis.

### Nested PCR

Only TV positive samples with sufficient DNA were included for analysis. Previously published MLST primers [9, 10] were used to amplify seven housekeeping genes: Tryptophanase (*tryp*), Glutaminase (*glut*), Alanyl tRNA synthase (*alts*), Family T2 asparagine-like threonine peptidase (P6) (*ft2a*), DNA mismatch repair protein (*dmrp*), Serine hydroxy methyltransferase (*shmt*) and Mannose 6-phosphate isomerase (*m6pi*). Inner Primers were modified by addition of adapters for Illumina sequencing and indexing. Q5 high-fidelity hot-start PCR master mix (New England Biolabs, USA) was used for the PCR amplification. Cycling conditions and primer sequences are presented in Supporting Information 1, S1 Table 1 and S2 Table 2. All samples were amplified in parallel along with TV genomic DNA (Reference G3, ATCC, USA), a TV negative clinical DNA sample from the same sample set and a water no template control (PCR grade H2O, Ambion, USA).

### Library preparation and sequencing

Each individual gene target amplicon was quantified using a Qubit 2.0 fluorometer and high-sensitivity dsDNA assay (Life Technologies, USA) and individually normalised to 10ng/µl and the 7 amplicons pooled by patient ID, in equal volume. Indexed libraries were prepared using the Nextera XT Index Kit V2, set B, (FC-131-2002). 2 × 250 V2 paired-end sequencing was performed using an Illumina MiSeq, following manufacturer’s instructions. Base calling and trimming of adapter sequences were undertaken by the MiSeq Reporter Software. Paired-end reads were trimmed using Trimmomatic (v 0.40) to a minimum phred score of 64.

### Analysis of MLST types

StringMLST [15], a command line tool designed to determine MLST of an organism directly from genome sequencing reads, assigned loci and sequence types (ST), using *Trichomonas vaginalis* STs downloaded from PubMLST (*n* = 72) (last download end 2023). A further set of previously published STs [10] were manually checked for matches and new locus combinations assigned as a new ST.

### Epidemiological data analysis

All participants completed a validated behavioural questionnaire capturing sexual behaviour, socio-demographic and other relevant data (original and translated copies of the questionnaire are presented in Supplementary file 1 (FSW-ITSV2 English) [14]. Vaginal symptoms, such as pain, bleeding, discharge or burning, together with age, number of clients, number of cities worked in the last 6 weeks and weekly earnings were analysed for individuals working in locations A, B and C, alongside the ST data. Data were expressed as frequencies and percentages for categorical variables and medians with interquartile ranges (IQR) for continuous variables. Comparisons of categorical and continuous variables were done using Chi-squared (χ2) and Kruskal-Wallis test, respectively. Analyses were performed using IBM^®^ SPSS^®^ version 24 and R version 4.1.3.

### Analysis of clonal complexes and population structure

TV MLSTs were analysed together with the 72 STs available on PubMLST by generating putative ‘clonal complexes’ (CCs), using the global optimal eBURST (Based Upon Related Strain Types (goeBURST) algorithm [16], with PHYLOVIZ 2.0 software. Generation of CCs was based on single locus variants (SLVs), i.e. STs that only differ by one allele type. Stringency factors for CC generation were iteratively relaxed, by changing the number of locus variant differences required to establish membership of a CC to test the stability of CCs produced.

The Bayesian clustering program STRUCTURE 2.3.3 [17] was used in combination with previously described allele frequencies [17] to estimate numbers of putative ancestral populations (*K)* represented by the sequenced TV strains, using an admixture model. STRUCTURE was run 10^5^ times for each of 10 K values (K = 1–10) with a burn-in period of 10^5^ iterations.

To estimate an adequate sample size for future studies, to better the understand the extent of diversity observed, a haplotype accumulation curve was generated using R version 4.6.1. using the package HACSim (Haplotype Accumulation Curve Simulator) [18].

## Results

Of the FSWs and NSW VVS samples, 58/250 (23%) and 1/250 (0.4%) were positive for TV by PCR respectively; 15 of 58 (25.9%) FSW TV positive samples were excluded due to insufficient volume or amplification failure of one or more of the 7 gene segments. Successful amplification from the single TV positive NSW sample, the FSW samples and the positive control resulted in 45 going forward for sequencing, with all but one, from an FSW, providing sequence data. A further six from FSW samples were omitted from analysis due to insufficient reads for at least one of the 7 gene loci, resulting in 38 MLST sequences being analysed.

### Clinical characteristics for *T. vaginalis* positive FSWs and NSWs

All participants from location A were BBWs, only one participant from location B was an SBW, whilst all participants from location C were SBWs. BBWs were younger than SBWs (median age 28 vs. 42 years respectively; *p* < 0.001). Among those positive for TV, BBWs had a higher number of clients per week than SBWs (median 30 (IQR 15–50) vs. 10 (IQR 5–30); *p* = 0.006) and had worked in more cities in the previous 6 months (median 5 (IQR; 0–20) vs. 1 (IQR; 0–7) respectively. As previously reported, among FSWs there was higher TV prevalence among SBWs (23/45) compared to BBWs (35/205) (51% vs. 17%, *p* < 0.001).

### Sequence type analysis

Of the 38 samples providing usable sequence data, 32 FSWs, 1 NSW and 1 positive control were assigned STs with a full set of 7 loci numbers using *stringMLST*. For the 32 FSW samples analysed, 17 (53.1%) were BBWs and 15 (46.8%) were SBWs.

The positive control and 3/33 (9.1%) non-control samples corresponded to previously documented STs. Of the remainder, 30 were new STs, 27 were identified once and two twice. Allele frequencies are presented in, Supporting Information 1, S3 Table 3.

**Fig. 1:**
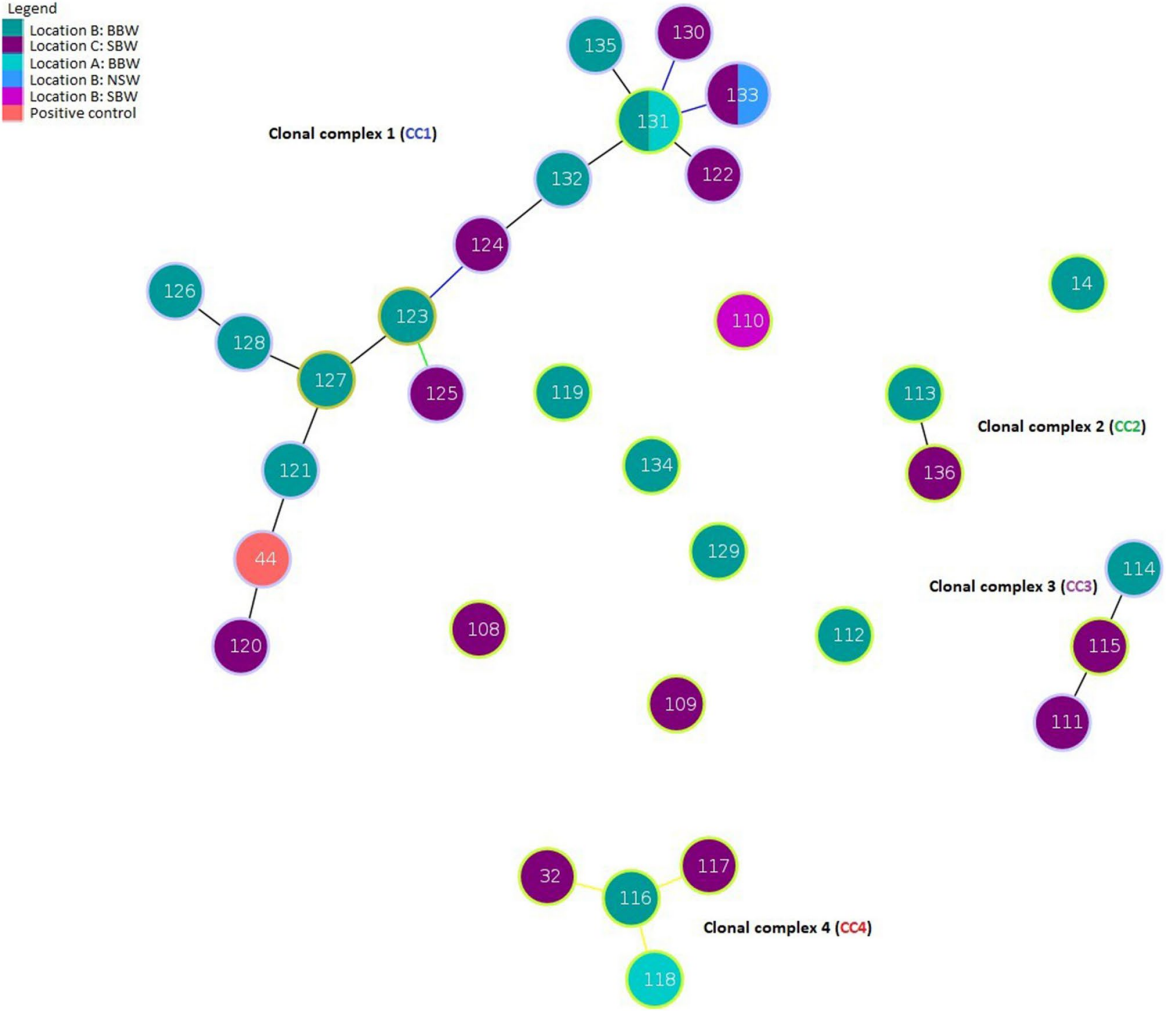
Initial *goeBURST* analysis of STs identified from samples in this study using *stringMLST.* All circles represent an ST, larger circles (131 and 133) correspond to that ST belonging to two individuals. Two FSWs represented by ST-131 were BBWs from location A and B, whilst the 133 represented the only NSW recruited from location B and a SBW from location C. Lines connecting each ST within CCs represent the rules applied using the goeBURST algorithm to link those two STs. Black lines refer to no recourse to tie break rules, blue shows a link corresponding to tie break rule one 1 and yellow refers to tie break rule 4 or 5 (frequency found on the data set and ST number, respectively) [16]

In an initial analysis, using only the STs generated in the 33 samples from this study alone, goeBURST identified four CCs, and eight isolated nodes, that is an isolated ST not in a CC (Fig. 1). The most populated complex, CC1, comprised of 15 SLV nodes, representing 17 participants and the positive control sample. The two participants assigned to ST-131 were BBWs recruited from location A and B, but lived in location C and B, respectively. The five nodes directly linked with ST-131 represent a mixture of three SBWs, two BBWs and the only NSW included in this study. The next largest CC was CC4, with ST-116 (location B) corresponding to a BBW, linked to two STs from two SBWs (location C and A). The third largest CC was CC3, that consisted of two SBWs and a BBW from a separate location; finally, CC2 included an SBW and a BBW from separate locations. When comparing membership to a CC or individual nodes, the STs of the 13 BBWs were found within CCs 1–4 compared to 11 of the SBWs (*p* > 0.05). Most FSWs included in the *goeBURST* analysis reported one or more vaginal symptoms (vaginal discharge, dyspareunia and/or burning in and around the vagina).

There was no difference in whether symptoms were reported by TV positive BBWs (82.4%; 14/17*) cf.* SBWs (60.0%; 9/15) (χ ^*2*^ *p* > 0.05).

**Fig. 2:**
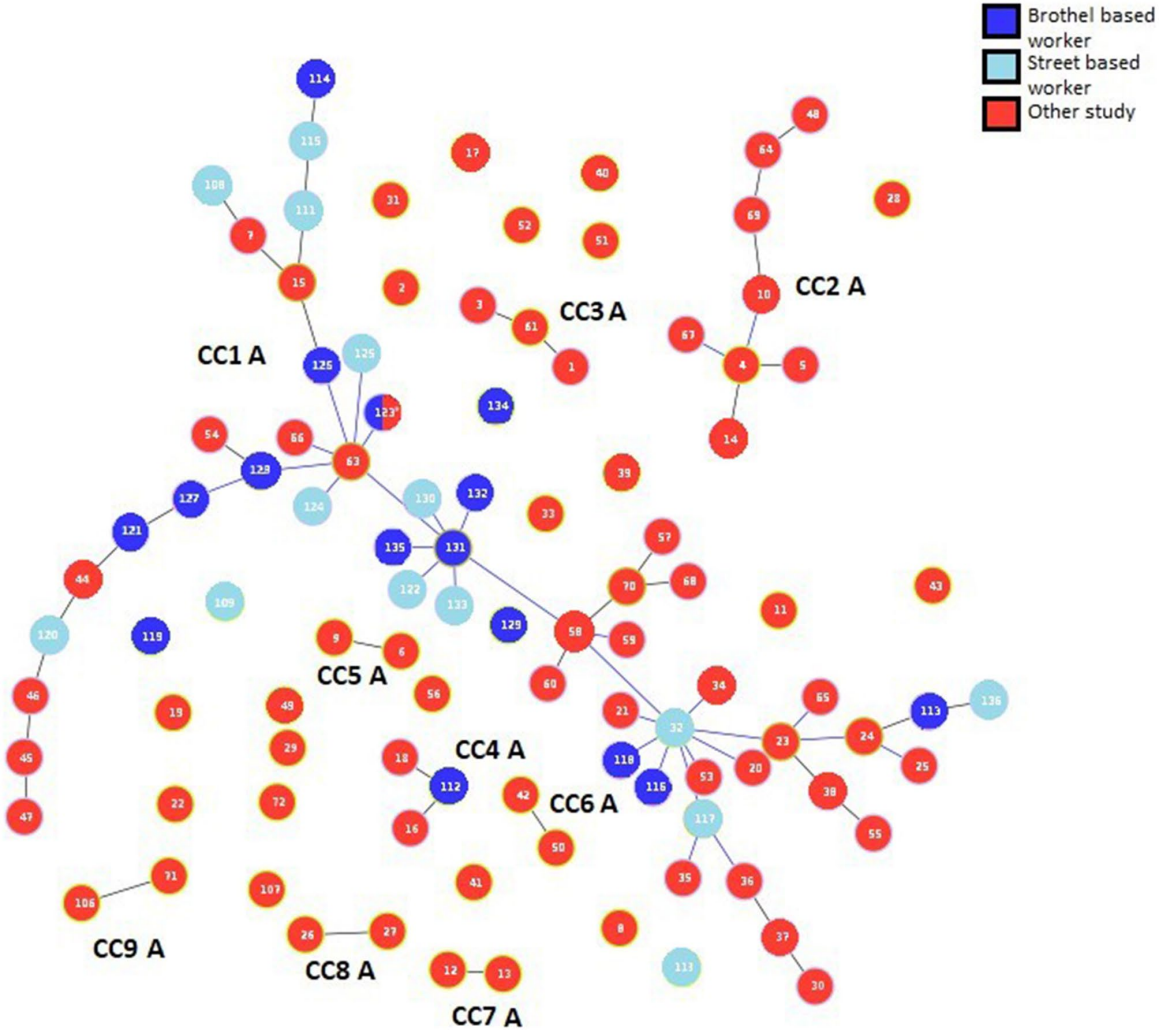
goeBURST analysis with samples reported from studies and stored within PubMLST. Samples were combined from this study and any available in PubMLST to understand how connected each CC would be when combined with a larger data set. As previously described, from the Ecuador dataset ST-131 and ST-133 both represent two individuals. In the larger dataset, 9 STs are represented by more than one individual (17, 32, 34, 37, 38, 39, 44, and 58). The samples ST-62 from Spain and ST-123 from Ecuador are represented by the same loci but only labelled as 123* in this diagram. All strains represented by STs here were collected from women between 1936–2021 from the USA, UK, Spain, Austria, and Ecuador. All non-Ecuador samples are presented in red

The 72 STs in pubMLST represented 83 cultured TV samples previously collected from females across north America and Europe (USA (*n* = 65), UK (*n* = 2), Austria (*n* = 1) and Spain (*n* = 15)). When we combined the 33 STs for this study with those available from PubMLST (Fig. 2), *goeBURST* revealed 9 clonal complexes and 24 individual nodes. Altering the stringency parameters of the *goeBURST* algorithm did not change the composition of the CCs, i.e. relaxing of the tie-break rules did not increase the number of STs introduced into the clonal complexes. In three of the four CC’s majority of STs are linked by a black line, thus tie break rules in goeBURST were not needed [16]. One CC was connected by yellow lines referring to the use of tie break rule 4 or 5, which refers to frequency found on the data set and ST number, respectively [16]. The colours of the lines from black to yellow indicate a decrease in confidence or certainty of this connection. In this combined analysis, 83.3% of STs assigned to FSWs became part of a large clonal complex, CC-1 A. Only 5 STs from our study fell outside of CC-1 A, with two BBWs and two SBWs being solitary nodes while another ST from a BBW, was part of CC-4 A.

All samples collected in our study were connected to isolates from either USA or Spain in the larger dataset, with none connected to the UK or Austrian STs.

**Fig. 3:**
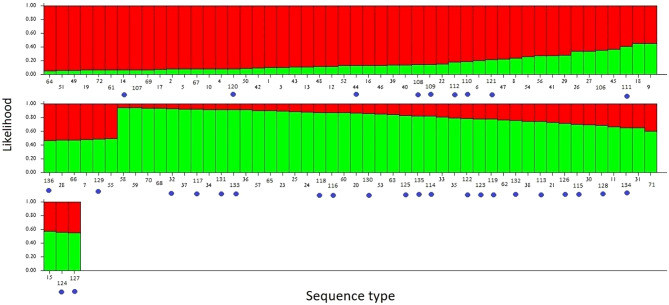
Two type population structure as determined by STRUCTURE. Type 1 I represented in red, and type 2 represented in green. Y-axis scale represents ancestry likelihood. Each bar represents an ST and the likelihood of ancestry to either type 1 (red) or type 2 (green). Samples with at least 0.8 (80%) ancestry to one of these population groups were used as a cut off to assign STs to that group, any of which had less than 0.8 ancestry were classed as unassigned samples. Blue circles represent STs from the Ecuador sample set, any STs without a blue circle represent samples taken from PubMLST

**Fig. 4:**
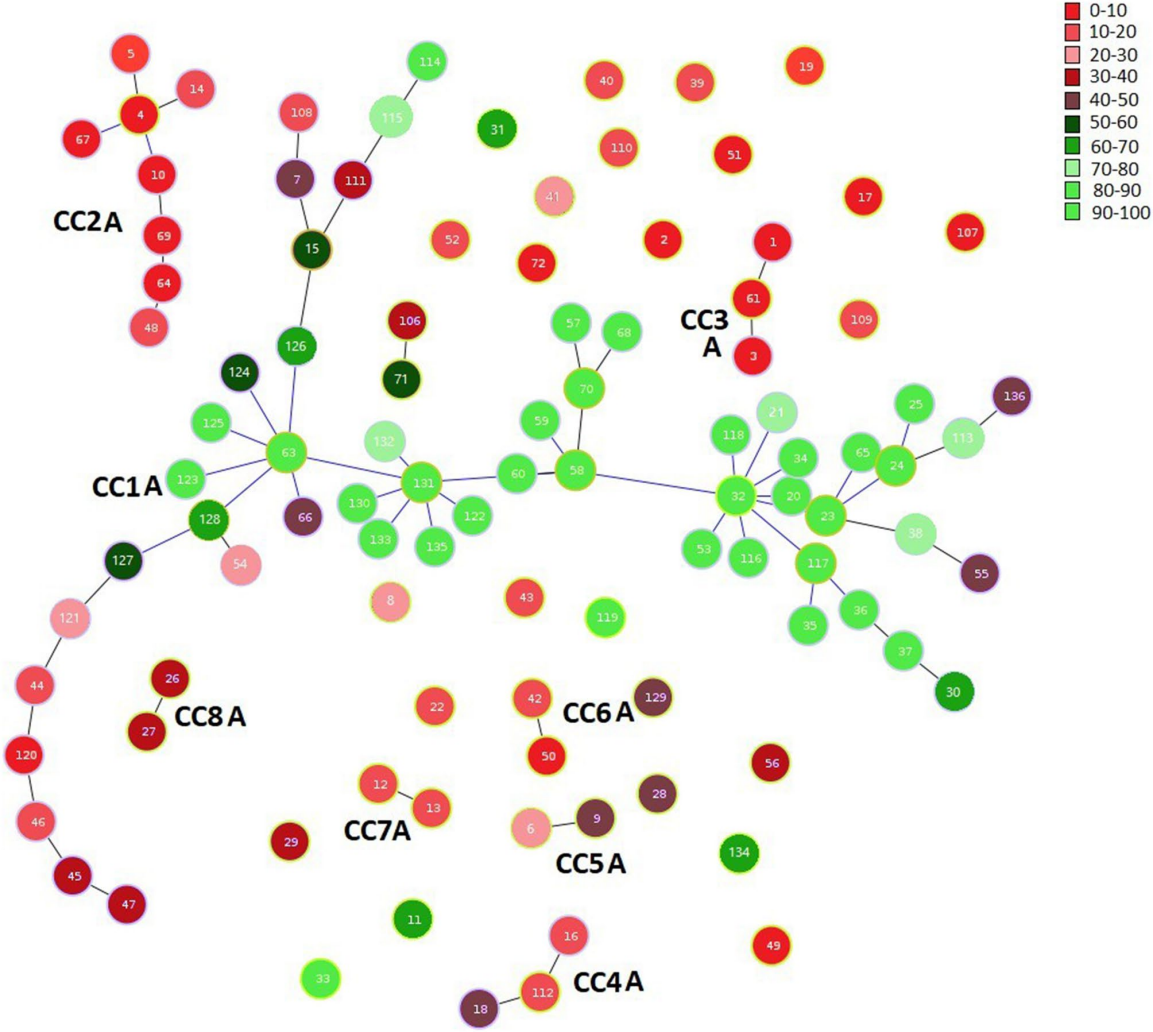
goeBURST of TV samples collected from FSWs and reported previously, coloured by reported ancestry to a subpopulation. In this instance all STs are coloured according to percentage (%) likelihood of their assignment to Type I or II populations

STRUCTURE analysis revealed a two-cluster ancestral population structure having the greatest log probability, with a default assignment to either type I or II population if they had at least 80% ancestry with that subpopulation (Fig. 3). Overall, 25.0% (8/32) of TV samples obtained from FSWs were assigned to Type I, 53.1% (17/32) to Type II and 21.9% (7/32) were unassigned. There was no difference between type I, II or unassigned (*p* > 0.05) by brothel or street-based sex worker status.

When population structure was mapped onto membership of clonal complexes, a relatively large ‘homogenous core’ of samples in CC-1 A were assigned to type II. The 20 STs peripheral to this homogenous core were linked to it in decreasing likelihood of type II content (Fig. 4).

Interestingly from the analysis without the international strains (Supporting Information 1, S1 Figure 1) the topological relationships of ST-131 (CC1) and ST-116 (CC4), and their linked nodes were largely preserved at the centre of CC-1 A, suggesting perhaps a more credible transmission link between individuals, whereas CC2 and 3 were separate to CC-1 A with different ancestral population probabilities.

Neither BBWs or SBWs from our Ecuadorian samples appeared to be more likely to be members of this homogeneous core, nor was there any obvious difference between recruitment location for the FSWs included in CCs or as individual nodes. Outside of CC-1 A, smaller CCs or single nodes were either ‘unassigned’, i.e. having < 80% ancestry or were assigned to Type I, consistent with clonal clusters largely being represented by a single type.

Use of HACSim generated 7 simulations of possible required sample sizes for future epidemiological studies. Based on the current data stored in PubMLST, combined with the data generated from this study, 97 individuals were represented by 91 haplotypes. The estimated sampling sufficiency for 95% haplotype recovery was 272 (271.31 -272.69) which would require a further 175 individuals to be sampled, after which the added benefit of sampling plateaus, Figure S2 (Supplementary information) is a graphical representation of this output.

## Discussion

We used a modified TV-MLST analysis for identifying strain diversity to explore its potential for sexual network identification, in a high TV-prevalence FSW population from Ecuador. We did this because of the technical and cost challenges of TV WGS. Overall, we demonstrated a high level of allelic diversity in TV DNA from different samples, despite the small number of samples sequenced.

This finding was in line with previous MLST studies from the UK and Australia reporting high numbers of new genotypes [11, 13]. As a means of validation, we combined our data with archived global ST data, enabling us to contextualise our large number of new STs within a previously collected dataset.

We analysed the ST allelic data using the global optimal eBURST algorithm separately and collectively with a set of international STs derived from previous studies, to identify clonal complexes, which are a putative description of TV associated sexual networks. When modifying the analysis by overlaying log probabilities of membership to ancestral populations, 8 clonal complexes and 24 individual STs, separate from any complex, were revealed. The former included a large clonal complex containing the majority of STs from our FSWs, interspersed with the international strains from Spain and South America, with a core of those with the same ancestral probability. In this core complex, two small clusters of FSW STs retained their internal topological relationship in the combined analysis, compared to an initial separate analysis containing only the Ecuador samples, and had the same ancestral population, one of which also contained a sample from a non-sex-worker. Two other such ‘preserved’ but smaller CCs appeared to have different ancestral populations. The preservation of the internal topology of CCs between some of these highly mobile FSWs with a common ancestral population structure suggests that this simplified stringent allelic identification and analysis approach, with lower cost and more technical feasibility compared to WGS, may have potential for TV associated sexual network identification and should be further evaluated. Increasing confidence in these findings particularly would require more stringent epidemiological validation as well as studies comparing such a method to WGS of TV.

Given that FSWs and their clients may often be important to understanding global spread of some STIs [19], it is perhaps unsurprising that TV genotyped from samples taken from highly sexually active and mobile sex workers from Ecuador would show high levels of genetic diversity and links to previously identified STs from Spain. TV has always shown high genetic heterogeneity globally when analysed using MLST and genotyping studies commonly report the presence of two distinct populations, type 1 and 2 [10, 11, 13]. In this study, we also identified two population types in similar numbers, which we also labelled types 1 and 2. In contrast to a previous study [10], which described STs assigned to type 2 being associated with individuals reporting a higher number of sexual partners in the past 6 months, we found no association between membership to a CC, subpopulations and numbers of clients reported or vaginal symptoms.

SBWs in this study were all living and working in the same location, possibly facilitating circulation of the same strains within a locale. Infection of TV among highly mobile FSWs is unlikely to be contained in one location, perhaps enabling transmission of infection between sex worker and non sex worker communities. It is also possible the persistent nature of TV infections can contribute to this effect, particularly given the high prevalence of asymptomatic infection, older age of women in this study and the lack of routine testing in Ecuador. For example, samples excluded from this analysis due to poor amplification all had a high Ct value (> 36). Of these, over 60% were collected from SBWs, possibly indicating a previously cleared infection, remnant DNA or a very low parasitic load, perhaps due to chronic infections. Persistent or chronic TV has been shown to be frequently associated with older age, possibly linked to altered mucosal immunity and microbiome changes such as loss of *Lactobacillus spp.* associated with menopause [20].

Data generated from previous studies have not been uploaded to the online open access repository PubMLST, and thus only 72 validated ST profiles are available, despite more than 120 additional STs being described in the literature [10, 11, 13]. This has resulted in many studies reporting overlapping STs numbers, but with different loci assignments recorded. We did not confirm the STs identified in this study against all other studies in the literature. There are, however, only few studies genotyping TV and this is the first report from South America. In comparison to frequently investigated organisms, such as *Neisseria spp*., there are over 17,500 STs available in PubMLST, emphasising the need for further TV genotyping studies. It was estimated that a further 175 samples would be required to understand the possible extent of diversity for TV (Figure S2, Supporting information). However, this was calculated based only on the data stored in PubMLST and not considering the other STs identified through the few MLST studies previously conducted and so may be inaccurate. Until affordable and accessible sequencing and bioinformatic methodologies are developed that can more easily resolve the TV genome, MLST remains a possible tool for discriminating strains for epidemiological studies and investigation.

### Limitations

There were several limitations to this study. Firstly, we were unable to use parasitic culture for these samples making it difficult to validate the genotypic profile of these infections while the low resolution of MLST restricted our ability to investigate mixed infections. However, it is noteworthy that culture-based methods may over represent certain strains that are better adapted to in vitro conditions [21].

Without WGS of the same samples, it was not possible to validate whether this method was able to discriminate highly similar or closely related strains. Presence of mixed infection, i.e., two strains present in one sample, was also not investigated in this study. In the absence of parasite culture, identification of mixed infections when using WGS, for example, is typically achieved by looking at numbers of heterozygous base calls. However, definitions of how to classify a heterozygous locus or allele can vary widely between studies [22] and determining cut-offs is best guided by availability of reference databases with multiple strains, which is currently not possible for TV. Secondly, we were also unable to identify any other features of the parasites such as co-colonisation with TVV, *M. hominis* or *Candidatus Mycoplasma girerdii*, which have all been implicated in TV pathogenicity [23–26]. We identified only one positive sample in the NSW population which limited our ability to investigate possible transmission events between different risk groups. Finally, the sample size of this study was small. As there are very limited data globally for TV and particularly from South America, it is difficult to establish how representative these data are.

## Conclusion

In conclusion, TV MLST was used on samples collected in Ecuador from both brothel and street-based sex workers, revealing diverse, including 31 new STs. The majority of STs were members of a large clonal complex that apparently had no link to any specific working location. Direct genotypic links between some STs among FSWs, in this clonal complex, were preserved following re-analysis after including a larger set of globally available archived STs. This suggests TV-MLST may have potential use as an aid to sexual network identification.

## Supplementary Information

Below is the link to the electronic supplementary material.

Supplementary Material 1

Supplementary Material 2

Supplementary Material 3

## Data Availability

Sequence data have been submitted to the European Nucleotide Archive database (https://www.ebi.ac.uk/ena/browser/home) with accession number PRJEB82350.

## Abbreviations

MLST: Multi locus sequence typing
TV: Trichomonas vaginalis
CC: Clonal complex
FSW: Female sex worker
ST: Sequence type
goeBURST: Globally optimal based upon related sequence types
