# Supplementary material for: Trichomonas vaginalis strain diversity among female sex workers in Ecuador using DNA sequence-based typing
Source: BMC Infect Dis. 2025 Dec 4;26:18. doi: 10.1186/s12879-025-12185-7 (PMC12781251; doi:10.1186/s12879-025-12185-7)
Supplement: Supplementary file 1 — Supplementary Material 1 [file 12879_2025_12185_MOESM1_ESM.pdf]

Machine Translated by Google

GAR-ITSV2

PARTICIPANT CODE *

CITY/TOWN SURVEY *

- Quinindé
- Las Golondrinas

Socio-epidemiological component

DATE OF BIRTH (DD, MM, YY) *

yyyy-mm-dd

BELONG TO ANY FSW ASSOCIATION *

- Yes
- No

CITY OF BIRTH *

CITY WHERE YOU LIVE MOST OF THE TIME *

HOW LONG HAVE YOU BEEN LIVING IN THE CITY YOU INDICATED? YEARS *

ARE YOU TEMPORARILY LEAVING THE CITY WHERE YOU LIVE? *

- Yes
- No

INDICATE THE REASON WHY YOU FREQUENTLY LEAVE THE CITY *

- Sex work
- Other work
- Studies
- Health
- Visit family or friends
- No answer

IN THE LAST 6 MONTHS, APPROXIMATELY, HOW MANY DIFFERENT CITIES HAVE YOU WORKED IN? *

AT WHAT AGE DID YOU START AS A SEX WORKER? *

SINCE YOU STARTED, HOW MANY TIMES HAVE YOU STOPPED WORKING? *

USUALLY, HOW MANY DAYS A WEEK DO YOU DO SEX WORK? *

APPROXIMATELY HOW MANY DIFFERENT CLIENTS DO YOU HAVE IN A WEEK? *

APPROXIMATELY HOW MUCH MONEY DO YOU EARN IN A WEEK? *

OF WHAT YOU EARN, WHO DO YOU SUPPORT FINANCIALLY ON A REGULAR BASIS? *

- You keep it
- To your partner
- To his protector/pimp
- To his family
- Debt payments
- No answer

WHAT YOU EARN PER WEEK IS ENOUGH TO SAVE *

- Yes
- No
- No answer

DO YOU CURRENTLY HAVE ANY OTHER JOB OTHER THAN SEX WORKER? *

- Yes
- No

WHAT IS THIS JOB? *

- Domestic worker
- Trader
- Agriculture
- Public or private employee

ARE YOU THE HEAD OF HOUSEHOLD? *

- Yes
- No

YOUR HOME IS: *

- Own
- Leased
- From relatives
- In exchange for the work

LET'S TALK ABOUT YOUR EDUCATION AND YOUR HOME. WHAT LEVEL OF EDUCATION DID YOU COMPLETE? *

- Illiterate
- Primary
- Secondary
- Superior

WHAT YEAR DID YOU PASS?

AT WHAT AGE DID YOU STOP STUDYING?

ARE YOU CURRENTLY STUDYING? *

- Yes
- No

Let's talk about your habits

IN THE LAST MONTH, HAVE YOU CONSUMED ALCOHOLIC BEVERAGES? *

- Yes
- No
- No answer

WHAT TYPE OF ALCOHOLIC BEVERAGE DID YOU DRINK? (WRITE THE MOST COMMON ONE)

DO YOU DRINK ALCOHOLIC BEVERAGES WHILE WORKING?

- Yes
- No
- No answer

Machine Translated by Google

WHAT ALCOHOLIC DRINK DO YOU DRINK WHILE WORKING?

HOW OFTEN DO YOU DRINK ALCOHOLIC BEVERAGES WHILE WORKING? *

- Always
- Almost always
- Occasionally
- Never

IN THE LAST MONTH, HAVE YOU SMOKED TOBACCO?

- Yes
- No
- No answer

DO YOU SMOKE TOBACCO WHILE WORKING?

- Yes
- No
- No answer

HOW OFTEN DO YOU SMOKE TOBACCO WHILE WORKING?

- Always
- Almost always
- Occasionally
- Never

HAVE YOU TRIED ANY KIND OF DRUGS IN YOUR LIFE? *

- Yes
- No
- No answer

IN THE LAST 3 MONTHS, HAVE YOU USED? *

- Marihuana, furthermore, cannabis
- Heroin
- Cocaine
- Ecstasy/Tacks
- I have not consumed
- No answer

Machine Translated by Google

IN THE PAST SIX MONTHS, HAVE YOU TAKEN TRANQUILIZERS OR SLEEPING PILLS?

- Yes
- No
- No answer

Sexual behaviors

*

LET'S TALK ABOUT YOUR CUSTOMERS. HOW DO YOU DETECT THE HEALTH STATUS OF YOUR CUSTOMERS?

- By general appearance
- By the smell
- By his penis
- By his face
- Directly asking questions
- Indirectly talking
- I can't know
- No answer

*

WE HAVE LEARNED THAT FEMALE SEX WORKERS HAVE REGULAR CLIENTS, NOT REGULAR CLIENTS, LET'S TALK ABOUT THEIR REGULAR CLIENTS. HOW MANY REGULAR CLIENTS DO YOU HAVE?

....

*

SEXUAL RELATIONS CAN BE ORAL, VAGINAL AND ANAL. LET'S TALK ABOUT THE TYPE OF SEXUAL RELATIONS AND THE USE OF CONDOMS. WHEN YOU HAVE ORAL SEX WITH YOUR REGULAR CLIENTS, HOW OFTEN DO YOU USE A CONDOM?

- Always
- Almost always
- Occasionally
- Never
- Do not practice oral sex

*

WHEN YOU HAVE VAGINAL SEX WITH YOUR REGULAR CLIENTS, HOW OFTEN DO YOU USE A CONDOM?

- Always
- Almost always
- Occasionally
- Never

WHEN YOU HAVE ANAL SEX WITH YOUR REGULAR CLIENTS, HOW OFTEN DO YOU USE A CONDOM? *

- Always
- Almost always
- Occasionally
- Never
- Do not practice anal sex

THE LAST TIME YOU HAD PENETRATIVE SEX WITH A REGULAR CLIENT, DID YOU USE A CONDOM? *

- Yes
- No
- No answer

WHY DON'T YOU USE A CONDOM WITH YOUR REGULAR CLIENTS?

- I didn't have condoms on hand
- I had taken alcohol
- The customer paid more
- The client was very insistent
- I saw the client looking healthy
- I was attracted to the client
- The client had consumed alcohol
- I needed money
- I had my period
- I always use a condom
- No answer

NOW LET'S TALK ABOUT NON-REGULAR CLIENTS. WHEN YOU HAVE ORAL SEX WITH YOUR NON-REGULAR CLIENTS, HOW OFTEN DO YOU USE A CONDOM? *

- Always
- Almost always
- Occasionally
- Never
- Do not practice oral sex

WHEN YOU HAVE VAGINAL SEX WITH YOUR NON-REGULAR CLIENTS, HOW OFTEN DO YOU USE A CONDOM?

- Always
- Almost always
- Occasionally
- Never

WHEN YOU HAVE ANAL SEX WITH YOUR NON-REGULAR CLIENTS, HOW OFTEN DO YOU USE A CONDOM? *

- Always
- Almost always
- Occasionally
- Never
- Do not practice anal sex

THE LAST TIME YOU HAD PENETRATIVE SEX WITH A NON-REGULAR CLIENT, DID YOU USE A CONDOM?

- Yes
- No
- No answer

WHY DON'T YOU USE A CONDOM WITH YOUR NON-REGULAR CLIENT?

- I didn't have condoms on hand
- I had taken alcohol
- The customer paid more
- The client was very insistent
- I saw the client looking healthy
- I was attracted to the client
- The client had consumed alcohol
- I needed money
- I had my period
- I always use a condom
- No answer

WHAT IS YOUR CURRENT RELATIONSHIP STATUS?

- Without a partner
- With a stable partner and lives with this person
- With a stable partner and does not live with this person
- With casual partners

NOW LET'S TALK ABOUT SEX WITH YOUR PARTNER. WHEN YOU HAVE ORAL SEX WITH YOUR PARTNER, HOW OFTEN DO YOU USE A CONDOM? *

- Always
- Almost always
- Occasionally
- Never
- Do not practice oral sex

WHEN YOU HAVE VAGINAL SEX WITH YOUR PARTNER, HOW OFTEN DO YOU USE A CONDOM?

- Always
- Almost always
- Occasionally
- Never

*

WHEN YOU HAVE ANAL SEX WITH YOUR PARTNER, HOW OFTEN DO YOU USE A CONDOM?

- Always
- Almost always
- Occasionally
- Never
- Does not practice anal sex

*

WHEN WAS THE LAST TIME YOU HAD PENETRATIVE SEX WITHOUT USING A CONDOM WITH YOUR PARTNER?

- last week
- between 2 to 4 weeks
- between 1 to 3 months
- more than 3 months
- I always use a condom

*

WHY DIDN'T YOU USE A CONDOM THE LAST TIME WITH YOUR PARTNER?

- He doesn't use it with his stable partner
- There's no need
- I don't use it because I want it
- He doesn't know how to approach it
- I didn't have condoms on hand
- My partner doesn't like it
- We had taken alcohol
- I had my period
- No answer

*

IF YOU USE A CONDOM WITH YOUR PARTNER, WHO MAKES THE DECISION TO USE IT?

- You
- Your partner
- Both
- We don't use condoms

WHEN YOU USE CONDOMS, WHERE DO YOU FREQUENTLY GET THEM? WRITE DOWN WHAT IS USUAL *

- You buy it in a pharmacy or store.
- They deliver it to you at the health center or hospital
- At your workplace

IN THE PAST 6 MONTHS, HAS A CONDOM EVER BROKEN? *

- Yes
- No
- No answer

WHAT DO YOU DO WHEN A CONDOM BREAKS DURING SEX? *

- He puts another one on
- Goes to the doctor
- Not enough
- Take antibiotics
- Use creams or ovules
- Pregnancy test (on your own)
- Morning after pill
- Does nothing
- It has never happened to you
- No answer

HAVE YOU EVER USED A FEMALE CONDOM? *

- Yes
- No
- Does not know

WHERE DO YOU GET THE FEMALE CONDOM? *

- You buy it in a pharmacy or store.
- They deliver it to you at the health center or hospital
- At your workplace

DURING YOUR LAST SEXUAL INTERCOURSE, DID YOU USE LUBRICANTS (GLYCERIN, VASELINE, SALIVA, CREAM, OIL)? *

- Yes
- No
- No answer

Machine Translated by Google

LET'S TALK ABOUT GENITAL CLEANLINESS. IN THE PAST 6 MONTHS, HAVE YOU USED ANY OF THESE SUBSTANCES: *

CREMA DENTAL?

- Yes
- No

IN THE PAST 6 MONTHS, HAVE YOU USED ALCOHOL? *

- Yes
- No

IN THE PAST 6 MONTHS, HAVE YOU USED GENTAMAX? *

- Yes
- No
- Does not know

IN THE PAST 6 MONTHS, HAVE YOU USED CANESTEN? *

- Yes
- No
- Does not know

IN THE PAST 6 MONTHS, HAVE YOU USED TRIGENTA? *

- Yes
- No
- Does not know

HAVE YOU EVER BEEN FORCED TO HAVE SEX AGAINST YOUR WILL? *

- Yes
- No
- No answer

WHO WAS IT WITH?

- [ ] Your partner
- [ ] Customer
- [ ] An unknown
- [ ] Family members
- [ ] Friend
- [ ] Protector/pimp
- [ ] Police/military
- [ ] No answer

WAS A CONDOM USED IN THOSE INVOLUNTARY RELATIONSHIP(S)?

- ○ Yes
- ○ No
- ○ No answer

IN THE PAST 6 MONTHS, HAVE YOU HAD GROUP SEX (ORGIES) OR PARTNER SWAP? *

- ○ Yes
- ○ No
- ○ No answer

IN THE PAST 6 MONTHS, HAVE YOU USED SEX TOYS (VIBRATOR, RING, ETC.) IN YOUR SEXUAL RELATIONS? *

- ○ Yes
- ○ No
- ○ No answer

Let's talk about your gynecological-obstetric history.

AT WHAT AGE DID YOU HAVE YOUR FIRST SEXUAL EXPERIENCE WITH PENETRATION? (VAGINAL, ANAL OR ORAL) *

....

HOW MANY TIMES HAVE YOU BEEN PREGNANT? (INCLUDE PREGNANCIES AND ABORTIONS) *

....

HOW MANY NORMAL BIRTHS? *

....

HOW MANY CAESAREAN SECTIONS? *

....

HOW MANY ABORTIONS? *

....

Machine Translated by Google

HOW MANY LIVING CHILDREN DO YOU HAVE? *

AGE OF YOUR FIRST CHILD *

AGE OF YOUR LAST CHILD *

HOW MANY COMMITMENTS OR SEXUAL PARTNERS HAVE YOU HAD IN YOUR LIFE? *

HOW LONG AGO DID YOU HAVE A PAP Smear? *

- One year
- Two years
- More than three years
- It has not been done
- Don't know

IN THE PAST 6 MONTHS, WHAT METHODS HAVE YOU USED TO NOT GET PREGNANT? *

- [ ] No method
- [ ] Condom
- [ ] Withdrawal method
- [ ] Oral contraceptive
- [ ] Copper T (DIU)
- [ ] Injectable contraceptive
- [ ] Contraceptive patches
- [ ] Contraceptive implants
- [ ] Hysterectomy/tubal ligation
- [ ] Menopause
- [ ] No answer

WHEN WAS THE LAST TIME YOU WENT TO THE DOCTOR OR OBSTETRIST FOR A GYNECOLOGICAL CHECKUP? (YEARS) *

Background diseases

HAVE YOU EVER HAD DENGUE? *

- ◯ Yes
- ◯ No
- ◯ Do not know

HAVE YOU EVER HAD CHIKUNGUNYA? *

- ◯ Yes
- ◯ No
- ◯ Do not know

HAVE YOU EVER HAD ZIKA? *

- ◯ Yes
- ◯ No
- ◯ Do not know

HAS A DOCTOR OR OBSTETRICIAN EVER TOLD YOU IF YOU HAVE HAD PELVIC INFLAMMATORY DISEASE? *

- ◯ In the last month
- ◯ between 1-3 months
- ◯ more than 3 months
- ◯ never
- ◯ do not know that disease

IF YOU HAD PELVIC INFLAMMATORY DISEASE, WHAT TREATMENT HAVE YOU RECEIVED? *

- ☐ Pills/injections
- ☐ Creams and ovules
- ☐ Received nothing

THE TREATMENT FOR PELVIC INFLAMMATORY DISEASE WAS GIVEN BY: *

- ◯ Health Center/Hospital
- ◯ I bought on my own
- ◯ Friend or family member
- ◯ Primary Care Technician (TAP)

HAS A DOCTOR OR OBSTETRICIAN EVER TOLD YOU IF YOU HAVE HAD CHLAMYDIA? *

- ◯ in the last month
- ◯ between 1-3 months
- ◯ more than 3 months
- ◯ never
- ◯ do not know the disease

IF YOU HAD CHLAMYDIA, WHAT TREATMENT HAVE YOU RECEIVED? *

- [ ] Pills/injections
- [ ] Creams and ovules
- [ ] Received nothing

TREATMENT FOR CHLAMYDIA WAS GIVEN BY: *

- ○ Health Center/Hospital
- ○ I bought on my own
- ○ Friend or family member
- ○ Primary Care Technician (TAP)

HAS A DOCTOR OR OBSTETRICIAN EVER TOLD YOU IF YOU HAVE HAD GONORRHEA? *

- ○ in the last month
- ○ between 1-3 months
- ○ more than 3 months
- ○ never
- ○ do not know the disease

IF YOU HAD GONORRHEA, WHAT TREATMENT HAVE YOU RECEIVED? *

- [ ] Pills/injections
- [ ] Creams and ovules
- [ ] Received nothing

TREATMENT FOR GONORRHEA WAS GIVEN BY: *

- ○ Health Center/Hospital
- ○ I bought on my own
- ○ Friend or family member
- ○ Primary Care Technician (TAP)
- ○ No answer

HAS A DOCTOR OR OBSTETRICIAN EVER TOLD YOU IF YOU HAVE HAD TRICHOMONIASIS? *

- ○ in the last month
- ○ between 1-3 months
- ○ more than 3 months
- ○ never
- ○ Do not know the disease

Machine Translated by Google

IF YOU HAD TRICHOMONIASIS, WHAT TREATMENT HAVE YOU RECEIVED? *

- [ ] Pills/injections
- [ ] Creams and ovules
- [ ] Received nothing

TREATMENT FOR TRICHOMONIASIS WAS GIVEN BY: *

- ○ Health Center/Hospital
- ○ I bought on my own
- ○ Friend or family member
- ○ Primary Care Technician (TAP)
- ○ No answer

HAS A DOCTOR OR OBSTETRICIAN EVER TOLD YOU IF YOU HAVE HAD SYPHILIS? *

- ○ in the last month
- ○ between 1-3 months
- ○ more than 3 months
- ○ never
- ○ Do not know the disease

IF YOU HAD SYPHILIS, WHAT TREATMENT HAVE YOU RECEIVED? *

- [ ] Pills/injections
- [ ] Creams and ovules
- [ ] Received nothing

THE TREATMENT FOR SYPHILIS WAS GIVEN BY: *

- ○ Health Center/Hospital
- ○ I bought on my own
- ○ Friend or family member
- ○ Primary Care Technician (TAP)
- ○ No answer

HAVE YOU EVER HAD AN HIV TEST? *

- ○ Yes
- ○ No
- ○ No answer

Machine Translated by Google

WHAT WAS THE RESULT OF THE TEST? *

- Positive
- Negative
- I didn't find out
- I did not receive results

HOW LONG AGO WAS THE LAST HIV TEST? ANSWER IN MONTHS *

Current health status

ARE YOU CURRENTLY EXPERIENCING VAGINAL DISCHARGE? *

- Yes
- No

ARE YOU CURRENTLY EXPERIENCING PELVIC OR LOWER ABDOMINAL PAIN? *

- Yes
- No

DO YOU FEEL LIKE YOUR VAGINA IS BURNING? *

- Yes
- No

DO YOU FEEL LIKE THERE ARE LESIONS IN THE VAGINA? *

- Yes
- No

DO YOU FEEL LIKE THERE ARE SPOTS OR BUMPS ON YOUR VAGINA? *

- Yes
- No

DO YOU FEEL PAIN DURING SEX? *

- Yes, slight pain
- Yes, deep pain
- Yes, both
- No

Machine Translated by Google

WHEN WAS THE LAST TIME YOU TOOK MEDICATION ON YOUR OWN FOR VAGINAL DISCHARGE OR LOWER ABDOMEN PAIN? WHETHER SUPPOSITORIES, CREAMS, PILLS OR INJECTIONS *

- ◯ 1 week ago
- ◯ 1-4 weeks
- ◯ 1-3 months
- ◯ more than 3 months
- ◯ I have not taken treatment

WHAT MEDICINE DID YOU USE? *

- ◯ Pain tablets
- ◯ Injections
- ◯ SUPPOSITORIES
- ◯ Creams
- ◯ Antibiotics
