# Supplementary material for: Trichomonas vaginalis strain diversity among female sex workers in Ecuador using DNA sequence-based typing
Source: BMC Infect Dis. 2025 Dec 4;26:18. doi: 10.1186/s12879-025-12185-7 (PMC12781251; doi:10.1186/s12879-025-12185-7)
Supplement: Supplementary file 3 — Supplementary Material 3 [file 12879_2025_12185_MOESM3_ESM.docx]

1. Supporting information

| STEP | TEMP | TIME |
| --- | --- | --- |
| Initial Denaturation | 98°C | 30 seconds |
| 30 Cycles (outer round 1)  20 cycles (inner round 2) | 98°C  59.7°C  72°C | 10 seconds  30 seconds  50 seconds |
| Final Extension | 72°C | 2 minutes |
| Hold | 4–10°C | ∞ |

1. S1 Table 1: Nested PCR conditions for the MLST protocol

3

| Gene | Outer F primer | Outer R primer | Inner F primer | Inner R primer |
| --- | --- | --- | --- | --- |
| Tryptophenase (*tryp*) | GAGATGTTCAAG TATGCCGATG | CAAGCTGGACTG TGAAGTGG | CGTCAACATCG GTGGCTTCA | GCGACAGCG ACGACATTCA  T |
| Glutaminase  (*glut*) | AAACGCTGGTGC  CATTACAAC | AAGGTTCTTGCC  ACGGATTG | TCGTTAATAGTG  GGTAAGGACG | CCAAGTATAG  CTCCGCTGAC |
| Family T2  asparagine-like threonine peptidase (P6)  (*ft2a*) | AAGGCTGTTGAG CGTGGTGC | GGTGTTCCGCCT GTAGAAGTGC | GAACAGGAGC ACCAGCAGAA | TCTCTAGCAA CGCAGCCAA C |
| Alanyl tRNA  synthase (*alts*) | AAGGAGGCAGAA  GACAAGTGG | TGGAGCATACTTA  GGAATGTTAGC | TCTGTCCAGGA  TGGTGTCTT | ACGCCTTCCT  CCTTCATCTT |

| DNA mismatch  repair protein (*dmrp*) | GGACTATCCTAC GATGCTATTGAG | GTGACCAAATTTC GCACCAAG | TCAAGGATATG GAAATCATCG | GTCTTCCGTG CGGACAATTC |
| --- | --- | --- | --- | --- |
| Serine  hydroxymethyltra nsferase (*shmt*) | CAGGTGATACATT TATGGGATTG | GGCTATCTGTTC CTCCACTGAC | GCTGAGTGAAC GGTGGAACATT | GAAGATGAGG TCCTCCTTGA |
| Mannose 6-  phosphate  isomerase (*m6pi*). | TTACGGTTTTCGT CCATTTG | GACAACAACACC TCTTCCTGC | AGCCAGTTGGC TTCTGAGTT | AACAATTCCG CAAGCTGGG  AG |

4 S2 Table 2: Primer sequences for inner and outer primers.

5

| TRYP | GLUT | FT2A | ALTS | DMRP | SHMT | M6PI | Sequence  Type (ST) | New ST |
| --- | --- | --- | --- | --- | --- | --- | --- | --- |
| 1 | 3 | 6 | 9 | 5 | 1 | 2 | 118 | Y |
| 5 | 3 | 1 | 4 | 5 | 1 | 2 | 131 | Y |
| 5 | 3 | 1 | 4 | 5 | 1 | 2 | 131 | Y |
| 5 | 1 | 6 | 11 | 5 | 1 | 1 | 129 | Y |
| 5 | 3 | 4 | 4 | 5 | 1 | 2 | 135 | Y |
| 5 | 1 | 1 | 12 | 1 | 1 | 2 | 127 | Y |
| 5 | 3 | 1 | 11 | 1 | 1 | 2 | 134 | Y |
| 5 | 3 | 1 | 4 | 2 | 1 | 2 | 132 | Y |

| 1 | 1 | 1 | 12 | 2 | 1 | 2 | 112 | Y |
| --- | --- | --- | --- | --- | --- | --- | --- | --- |
| 2 | 1 | 1 | 12 | 1 | 1 | 2 | 121 | Y |
| 1 | 1 | 1 | 2 | 8 | 1 | 1 | **14** | N |
| 1 | 3 | 6 | 12 | 1 | 1 | 2 | 119 | Y |
| 5 | 1 | 1 | 9 | 5 | 1 | 2 | 126 | Y |
| 1 | 3 | 1 | 6 | 1 | 1 | 2 | 114 | Y |
| 5 | 1 | 1 | 12 | 5 | 1 | 2 | 128 | Y |
| 5 | 1 | 1 | 4 | 1 | 1 | 2 | 62/123 | Y |
| 1 | 3 | 1 | 2 | 6 | 1 | 2 | 113 | Y |
| 1 | 3 | 6 | 2 | 5 | 1 | 2 | 116 | Y |
| 1 | 1 | 1 | 2 | 9 | 1 | 2 | 108 | Y |
| 1 | 3 | 1 | 9 | 1 | 1 | 2 | 115 | Y |
| 2 | 1 | 1 | 3 | 8 | 1 | 2 | 120 | Y |
| 5 | 1 | 1 | 4 | 6 | 1 | 2 | 125 | Y |
| 5 | 1 | 1 | 4 | 2 | 1 | 2 | 124 | Y |
| 1 | 3 | 1 | 2 | 4 | 1 | 2 | 136 | Y |
| 1 | 3 | 6 | 6 | 5 | 1 | 2 | 117 | Y |
| 1 | 1 | 1 | 9 | 1 | 1 | 2 | 111 | Y |
| 5 | 3 | 1 | 6 | 5 | 1 | 2 | 133 | Y |

| 2 | 3 | 1 | 4 | 5 | 1 | 2 | 122 | Y |
| --- | --- | --- | --- | --- | --- | --- | --- | --- |
| 5 | 3 | 1 | 2 | 5 | 1 | 2 | 130 | Y |
| 1 | 3 | 6 | 4 | 5 | 1 | 2 | **32** | N |
| 1 | 1 | 1 | 2 | 1 | 1 | 3 | 109 | Y |
| 1 | 1 | 1 | 4 | 8 | 1 | 3 | 110 | Y |
| 5 | 3 | 1 | 6 | 5 | 1 | 2 | 133 | Y |
| 2 | 1 | 1 | 3 | 1 | 1 | 2 | **44** | N |

1. S3 Table 3: MLST sequence types and allele numbers as generated by stringMLST, for each sample
2. with sequencing results


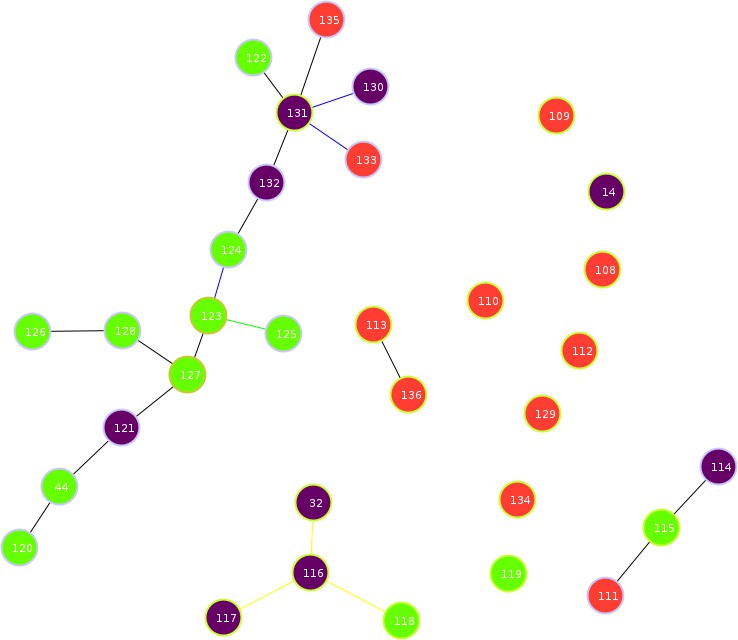


1. S1 Figure 1: Population types mapped onto goeBURST clustering of *T. vaginalis* STs. Colours
2. represent assignment to either type I (red), or type II (green). Any STs with mixed ancestry are
3. represented in purple.

11

12
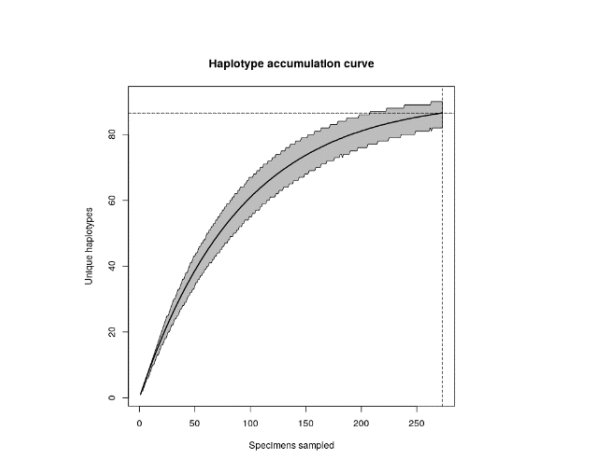


S1 Figure 2: Haplotype accumulation curve generated by HACSim in R, to estimate the number of additional samples required to better understand the distribution of genotypes in *T. vaginalis* samples.
